# Infection Dynamics and Host Biomarker Identification for Spotty Liver Disease in Chickens

**DOI:** 10.3390/vetsci12121136

**Published:** 2025-11-29

**Authors:** Helen James, Emily Gan, Robert J. Moore, Daniel M. Andrews, Thi Thu Hao Van

**Affiliations:** 1 School of Science, RMIT University, Bundoora, VIC 3083, Australia; helenjamestharakan03@gmail.com (H.J.); rob.moore@rmit.edu.au (R.J.M.); 2 Bioproperties Pty Ltd., RMIT University, Bundoora West Campus, Bundoora, VIC 3083, Australia; emily.gan@bioproperties.com.au (E.G.); dan.andrews@bioproperties.com.au (D.M.A.)

**Keywords:** spotty liver disease, layer hens, RNA sequencing, differential gene expression, egg production, biomarkers, hepatic metabolism

## Abstract

Spotty liver disease (SLD), primarily caused by *Campylobacter hepaticus* and (less frequently) by *Campylobacter bilis*, significantly impacts commercial layer hens by causing liver lesions, reducing egg production, and increasing mortality, meaning it can result in serious economic losses for farmers. This study explored the relationship between infection, liver dysfunction, and reproduction, aiming to identify host genetic markers for tracking SLD progression. Hens were orally inoculated with the *C. hepaticus* strain NSW44L and monitored over a seven-day period. Pathogen colonisation was quantified using qPCR across the liver, bile, caeca, spleen, and ovarian follicles, while liver lesions were scored and hepatic transcriptomes analysed using RNA-seq. *C. hepaticus* was detected in the liver, caeca, and spleen from one day post-inoculation (dpi) (1.44–1.68 log_10_ CFU/mL), appeared in bile by the third dpi (3.64 log_10_ CFU/mL), and reached the follicles by the fourth dpi (3.25 log_10_ CFU/mL). The highest bacterial loads were found in bile on days six and seven (up to 7.18 CFU/mL). Liver lesions were first observed on the fourth dpi, reaching their peak at the sixth and seventh dpi. Gene expression analysis in liver tissue revealed a notable downregulation of yolk-precursor and metabolic genes, such as prolactin receptor (PRLR), 7-dehydrocholesterol reductase (DHCR7), and malic enzyme 1 (ME1). In contrast, from days three to seven post-infection, there was significant upregulation of avidin (AVD), a biotin-binding protein, and versican (VCAN), which is linked to tissue remodelling and inflammation. These findings correlate with the disease’s progression from initial liver infection to widespread bacterial presence, suggesting value as host biomarkers for effective SLD monitoring and the development of targeted therapies.

## Simple Summary

Spotty liver disease poses a serious problem for commercial layer hens, leading to liver lesions, decreased egg production, and financial losses for farmers. The disease is primarily caused by the bacterium *Campylobacter hepaticus*. This study aimed to explore how the disease progresses in hens and to find host genetic markers that could help track its development. We infected hens and monitored them for seven days. Quantitative PCR (qPCR) showed that *C. hepaticus* appeared in the liver, caeca, and spleen at 1 day post-inoculation (dpi), in bile by 3 dpi, and in follicles by 4 dpi, with the highest loads in bile at 6–7 dpi (up to 7.2 log_10_ CFU/mL). Liver lesions were first visible on the fourth day post-infection and increased in number over the next three days. RNA analysis of liver tissue showed a decrease in in expression of genes related to egg production and an increased expression in those associated with inflammation and tissue changes. These insights into the disease’s progression could lead to better monitoring and control strategies, ultimately reducing economic losses for poultry farmers.

## 1. Introduction

Spotty Liver Disease (SLD) is an infectious disease of chickens that primarily affects laying hens in free-range production systems, causing significant mortality and egg-production losses [1,2]. SLD outbreaks are characterised by widespread hepatitis and a sudden drop in flock performance, often with up to 10–15% flock mortality and 35% reductions in egg output [1,2]. The disease was first reported in the United States during the 1950s [2], and since then, it has been reported in various countries, including Australia [1], Costa Rica, Germany and Eastern Europe [3], Jordan [4], the United Kingdom [5], and New Zealand [6]. It is now regarded as one of the most significant bacterial disease challenges for the egg industry, especially as the sector shifts toward cage-free management [7]. Despite its impact, the specific etiologic agent of SLD remained elusive until recent years.

In 2015, a novel *Campylobacter* was isolated from laying hens with spotty liver disease in the UK, and the same bacterium was soon recovered from Australian flocks and formally named *Campylobacter hepaticus* in 2016 [8,9]. Subsequent infection trials fulfilled Koch’s postulates by reproducing SLD in hens challenged with *C. hepaticus*, confirming *C. hepaticus* as the causative agent [10].

*C. hepaticus* is a motile, thermophilic Gram-negative bacterium with an unusually small genome (~1.5 Mb, 27–28% G + C) relative to other campylobacters, reflecting its specialised adaptation to the chicken hepatic system, including the liver, gallbladder, and bile [11,12]. It is difficult to culture but can be isolated from the bile and liver of diseased birds, where it often predominates as a near-pure infection [9]. Infected chickens develop characteristic multifocal necrotic lesions on the liver surface that give SLD its name, and the bacterium has also been detected in the intestinal tract of affected birds—suggesting an oral route of entry with subsequent hepatic colonisation [8,10]. In 2023, *C. bilis* was also confirmed as a cause of SLD, although it is less frequently reported compared to *C. hepaticus* [13]. Comparative studies of *Campylobacter* species offer valuable insight into *C. hepaticus* infection biology. *Campylobacter jejuni*, the most extensively studied avian-associated *Campylobacter*, shares key virulence features such as motility, bile resistance, epithelial adherence, and immune evasion [14,15,16]. Experimental evidence further shows that *C. jejuni* can establish persistent colonisation in the ceca and occasionally disseminate systemically [17,18]. Although *C. jejuni* primarily causes intestinal infection and only occasionally invades the liver, its well-characterised colonisation and immune evasion mechanisms may provide comparative insight into how *C. hepaticus* persists within the chicken gut–bile–liver axis.

Despite the identification of *C. hepaticus* and *C. bilis*, progress in understanding their basic biology and the mechanisms underlying SLD pathogenesis remains limited. Until now, its presence in the reproductive tract had not been demonstrated; this study provides the first evidence linking *C. hepaticus* dissemination beyond the liver to reproductive impacts in hens. Field outbreaks of SLD are consistently associated with substantial drops in egg production, implying that the infection can indeed disrupt normal nutrient allocation and reproductive processes; however, the mechanisms linking *C. hepaticus* colonisation and liver pathology to decreased laying performance remain undefined [19]. To date, most of the research on SLD has concentrated on pathogen characterisation, epidemiology, host immune responses, and alterations in the gut microbiota. [7,20,21,22,23]. However, the host’s hepatic transcriptomic response remains unexplored, leaving a critical gap in understanding how *C. hepaticus* perturbs liver function and contributes to metabolic and reproductive dysfunction.

To address this gap, we performed a time-course *C. hepaticus* experiment in commercial layer hens and sampled various tissues at sequential time points, from day 1 to day 7. RNA sequencing (RNA-Seq) was applied to profile hepatic gene expression changes during infection, enabling the sensitive detection of differentially expressed genes (DEGs) across the genome [12]. Analyses focused on DEGs linked to metabolic and reproductive functions. In addition, we examined how *C. hepaticus* colonises different organs by determining the timing and extent of bacterial establishment in the liver, bile, spleen, caeca, and ovarian follicles.

## 2. Materials and Methods

### 2.1. Animal Trial and Sample Collection

All animal procedures were approved by the Wildlife and Small Institutions Animal Ethics Committee of the Victorian Department of Economic Development, Jobs, Transport and Resources (Project 33.21, approved on 22 February 2022). *C. hepaticus* strain NSW44L was prepared as described in Eastwood et al. (2025) [20]. In brief, NSW44L was streaked onto horse blood agar (HBA) plates (Brucella broth supplemented with 1.5% agar (BD BBL™, Franklin Lakes, NJ, USA) and 5% defibrinated horse blood (Serum Australis, Melbourne, VIC, Australia)). *C. hepaticus* was then grown in Brucella broth supplemented with L-cysteine (0.4 mM), L-glutamine (4 mM), and sodium pyruvate (10 mM, Sigma-Aldrich, St. Louis, MO, USA) in tissue culture T75 flasks at 37 °C for 48 h in microaerophilic conditions at 37 °C and used directly for the challenge (5 × 10^9^ cfu/mL). One mL of this suspension was orally administered to each bird in the challenged group, while control birds received an equivalent volume of sterile culture medium. Eighty-four hens aged 23 weeks were used in the study and acclimated at the facility for two days prior to the commencement of the trial. They were randomly assigned to two groups: a challenged group and a control group (*n* = 42 for each group). Six birds from each group were necropsied per day for seven consecutive days to assess infection progression. Lesion severity was quantified by counting the number of visible lesions on the surface of the liver, then scored using a scoring system where 0 indicated no lesions; 1, 1–5 spots; 2, 6–20 spots; 3, 21–100 spots; 4, 101–1000 spots; and 5, >1000 spots [9]. Liver samples were excised and immediately immersed in RNAlater™ (Thermo Fisher Scientific, Waltham, MA, USA) in sterile Eppendorf tubes and stored at −80 °C until processing for RNA extraction and high-throughput RNA sequencing. Small pieces of liver tissues for *C. hepaticus* quantification were placed in separate sterile Eppendorf tubes and stored at −20 °C until analysed. No birds were excluded from the study as there were no unforeseen health issues, protocol deviations, or technical issues that warranted exclusion. All birds enrolled were included in the final analysis.

### 2.2. Bacterial Load Quantification

The bacterial load in liver, bile, ovarian follicles, spleen, and caecal content samples was determined using quantitative PCR (qPCR) with *C. hepaticus*-specific primers as described by Van et al. 2017 [24]. DNA was extracted using the Maxwell^®^ RSC instrument with the Maxwell^®^ RSC PureFood GMO and Authentication Kit (Promega, Madison, WI, USA), following the manufacturer’s instructions. Standard curves were generated from ten-fold serial dilutions of a culture of known colony-forming unit (CFU)/mL to enable quantification. Loads were expressed as CFU/mL equivalents. The detection limit of the assay was 10 CFU/mL.

### 2.3. Expression of Host Genes During C. hepaticus Infection

#### 2.3.1. RNA Extraction and Quality Control

Approximately 20–30 mg of liver tissue was homogenised, and total RNA was extracted using the Maxwell^®^ RSC 48 Instrument with the Maxwell^®^ RSC miRNA Tissue Kit (Promega, Madison, WI, USA) according to the manufacturer’s instructions. A DNase treatment step was included to remove residual genomic DNA. RNA concentration and purity were initially assessed spectrophotometrically by A260/280 and A260/230 ratios, and integrity was further evaluated on an Agilent 2100 Bioanalyzer using the RNA 6000 Nano Kit (Agilent Technologies, Santa Clara, CA, USA). Only samples meeting stringent quality criteria (≥1 µg RNA, RNA Integrity Number (RIN) > 7.0, rRNA ratio > 1.0, and concentration > 10 ng/µL) were selected for downstream library preparation.

#### 2.3.2. RNA Sequencing

Sequencing libraries were prepared from qualified RNA samples using the Illumina TruSeq Stranded mRNA protocol (Illumina, San Diego, CA, USA) according to the manufacturer’s instructions. Libraries were quantified, pooled, and quality-checked prior to sequencing. Paired-end sequencing (2 × 150 bp) was performed on the Illumina NovaSeq 6000 platform at Macrogen (Seoul, Republic of Korea). Forty samples were subjected to RNA-seq on post-infection days 3 through 7, with eight birds per day (four control and four challenged). A total of 136 GB of data was obtained, 3.4 GB per sample, corresponding to 11.5 million read pairs per sample.

#### 2.3.3. Bioinformatics and Differential Expression Analysis

Raw reads were processed and analysed using CLC Genomics Workbench (Qiagen, Hilden, Germany, version 24.0.1). Adapter sequences and low-quality bases were removed using default settings. The filtered reads were mapped to the *Gallus gallus* reference genome (GRCg7b assembly, Ensembl release 109) with strand-specific RNA-Seq parameters, and gene-level counts were generated using the corresponding Gene Transfer Format (GTF) annotation. Differential expression analysis was performed for each day (days 3–7 post-infection) by comparing the challenged and control groups. Genes with positive log_2_ fold change values were considered upregulated in the challenged birds relative to controls, whereas genes with negative log_2_ fold change values were considered downregulated. Genes associated with hepatic metabolism and reproductive function were prioritised for further investigation.

### 2.4. Statistical Analysis

Differential gene expression was computed in CLC Genomics Workbench (Qiagen, version 24.0.1) with FDR correction for multiple testing. Genes were considered significant at FDR < 0.05 and |log_2_ fold change| ≥ 1 [25]. All tests were two-sided unless stated otherwise. Bacterial load data obtained from quantitative PCR were expressed as mean ± standard error of the mean (SEM) to illustrate variability among biological replicates.

## 3. Results

### 3.1. Pathogen Load Dynamics Following C. hepaticus Challenge

**Figure 1:**
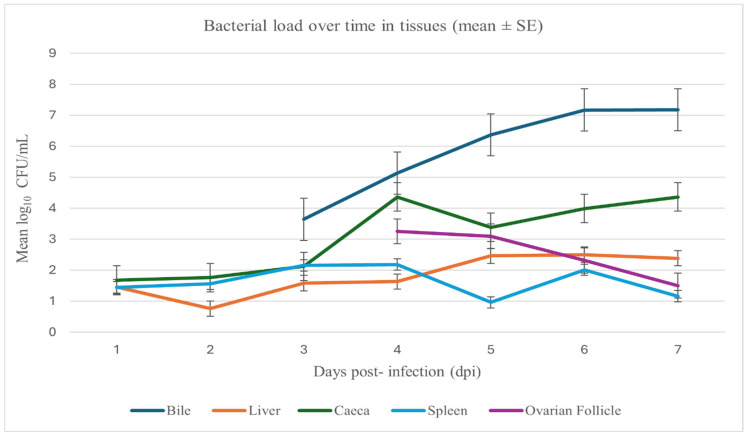
Bacterial loads of *C. hepaticus* in the liver, bile, spleen, caeca, and ovarian follicles of experimentally challenged hens from 1 to 7 days post-infection (dpi). Data points represent mean (log_10_ CFU/mL) ± standard error of the mean (SEM) for each tissue at each time point (*n* = 6). qPCR was performed in triplicate for each sample.

Quantification of *C. hepaticus* by qPCR revealed early, tissue-specific colonisation with progressive dissemination over 1–7 days post-infection (dpi) (Figure 1). At 1 dpi, low mean loads (log_10_ CFU/mL) were detected in the liver (1.45), caeca (1.68), and spleen (1.44), while presence in bile and ovarian follicles was not detected (ND). At 2 dpi, titres remained low in the liver (0.76), caeca (1.76), and spleen (1.56), with bile and ovary still ND. By 3 dpi, bile became positive (3.64), caeca and spleen showed modest increases (2.12 and 2.15, respectively), and liver remained low (1.58). At 4 dpi, all tissues were positive, with mean loads of bile 5.13, caeca 4.36, ovary 3.25, spleen 2.18, and liver 1.63. From 5 to 7 dpi, bile exhibited the highest and increasing loads (6.37 to 7.18), caeca continued to rise (3.38 to 4.36), ovary declined (3.09 to 1.50), liver remained low (2.46 to 2.38), and spleen stayed low with a transient rise at 6 dpi (0.96 to 2.01).

Collectively, these data indicate rapid post-challenge dissemination, with preferential and sustained colonisation of the biliary tract and caeca.

### 3.2. Development of Liver Lesion

Macroscopic lesions were absent from 1 to 3 dpi in all birds, so there was early pathogen colonisation without visible pathology (Table 1). The first signs of hepatic damage appeared at 4 dpi, with three of the six birds necropsied showing mild to moderate lesion scores of 1–3. By 5 dpi, four of the six challenged birds developed more pronounced pathology, including scores up to 4 (101–1000 spots). Lesion severity further increased at 6 dpi, where all six birds exhibited widespread hepatic foci, with scores ranging from 1 to 4. The trend continued at 7 dpi, with all six challenged birds presenting lesions, predominantly at severity levels 3–4. The onset of hepatic pathology occurred after bacterial colonisation had already been established in the liver and caeca and coincided with the detection of *C. hepaticus* in bile and spleen, and ovarian follicles.

### 3.3. DEGs Linked to Egg Production

Transcriptomic profiling was conducted on four control and four infected birds per day from 3 to 7 dpi. The analysis identified eight genes associated with egg production that were differentially expressed between challenged and control hens (Table 2). A coordinated reduction in genes essential for yolk precursor synthesis and lipid metabolism was observed during the early to mid-stages of infection. Prolactin receptor (PRLR) was significantly downregulated at days 3 and 6 post-infection, while enzymes supporting steroid and fatty-acid biosynthesis, including 7-dehydrocholesterol reductase (DHCR7) and malic enzyme 1 (ME1), were suppressed at days 4 and 6.

In contrast, several genes supporting nutrient uptake, calcium regulation, and extracellular matrix remodelling were consistently upregulated. Avidin (AVD) displayed strong induction from days 3 through 7, with a peak on day 5. Vitamin D receptor (VDR) expression rose sharply on day 5 and remained elevated on day 7. Very low-density lipoprotein receptor (VLDLR) significantly increased on days 5 and 6, coinciding with enhanced expression of versican (VCAN) across all five time points. Clusterin (CLU) showed a modest increase at days 6 and 7. Collectively, these results highlight infection-associated disruption of yolk precursor pathways, accompanied by induction of compensatory and stress-related responses.

### 3.4. DEGs Linked to Liver Function

Several genes associated with hepatic homeostasis and metabolism showed altered expression following *C. hepaticus* challenge (Table 3). Transcripts encoding acute-phase proteins were consistently elevated across the time course. Serum amyloid A (SAA) exhibited the strongest induction, with expression already increased on day 3 and reaching maximum levels on day 5. Orosomucoid-1 (ORM1) was also markedly upregulated, showing the highest fold change on day 4 and remaining elevated through day 7. Haemopexin (HPX) expression increased between days 4 and 7, with peak levels observed at day 6, while apolipoprotein A-IV (APOA4) was significantly upregulated on day 7. In addition, versican (VCAN) was consistently upregulated across all sampling days, with fold changes ranging from 1.19 to 1.87. Clusterin (CLU), a stress-inducible chaperone with hepatoprotective roles, was significantly up-regulated, peaking at 6 dpi (log_2_FC = 0.84) and remaining slightly elevated at 7 dpi (log_2_FC = 0.09), consistent with its role in hepatic stress responses.

In contrast, two metabolic genes demonstrated reduced expression late in infection. Phosphoglycerate dehydrogenase (PHGDH) was downregulated on day 7, and cytochrome P450 1A2 (CYP1A2) also showed lower transcript levels at this same point.

## 4. Discussion

### 4.1. Pathogen Load Dynamics in C. hepaticus Infection and Lesion Development

The infection characteristics of *C. hepaticus* resemble those of *C. jejuni*, a well-studied avian pathogen. In poultry, *C. jejuni* establishes persistent caecal colonisation aided by motility, chemotaxis, and immune evasion mechanisms [49]. Lipooligosaccharide modifications enhance bile resistance and facilitate successful colonisation in chicks [50]. Experimental infections show that *C. jejuni* may transiently reach systemic organs, including the liver and spleen, before clearance while maintaining high caecal loads [51]. Field studies confirm its persistence in commercial flocks and long-term caecal carriage—strongly influenced by gut microbiota composition—has been demonstrated specifically in laying hens [52,53,54,55,56]. These parallels suggest that the intestinal persistence and host–pathogen interactions described for *C. jejuni* may underpin the hepatic tropism of *C. hepaticus* in hens.

In this study, *C. hepaticus* showed a defined temporal colonisation pattern over seven days. The 7-day duration was chosen to capture the acute phase, as hepatic lesions typically develop within 5 days post-infection and peak within the first week. Nonetheless, a longer study could provide additional insight into the dynamics of infection [24]. Bacteria were detectable in the liver and caeca from 1 to 2 dpi, whereas bile colonisation was delayed until about day 3, suggesting initial intestinal establishment followed by hepatic invasion and subsequent biliary spread. Early liver detection supports the known invasiveness of *C. hepaticus* and the faecal-oral transmission route [21]. The gut may serve as the initial reservoir and a continuous source for liver reinfection.

By 3 dpi, bile samples became positive, indicating later gall bladder involvement. The absence of detectable *C. hepaticus* in bile during the first two days, and in ovarian follicle samples during the first three days, suggests that the bacteria were either absent or present at levels below the detection limit of the assay. Although colonisation of the liver was evident from 1 dpi, visible hepatic lesions were not observed until 4 dpi, when three birds displayed miliary spots (lesion scores of 1–3). Lesion prevalence and severity increased at 5–7 dpi, with most birds displaying moderate to severe pathology (scores 3–4). Despite low hepatic bacterial loads (≈1–2 log_10_ CFU/mL), severe lesions developed from 4 dpi onwards, suggesting that liver injury results primarily from toxin-mediated or immune-driven processes rather than bacterial accumulation, consistent with immunopathological mechanisms in *C. jejuni* [14,15,16]. This temporal relationship indicates colonisation alone is insufficient for pathology; lesions likely require sustained presence and host inflammation, with liver involvement preceding biliary colonisation.

By the late infection stage (days 6–7), the highest bacterial loads occurred in bile, even surpassing caecal titres, highlighting the gall bladder as a key niche for *C. hepaticus* [2]. Notably, despite peaking in bile, *C. hepaticus* remained consistently present in the caeca throughout the trial [24]. This observation aligns with reports of *C. hepaticus* persisting within the intestinal tract and caeca of experimentally infected hens [21]. Persistent colonisation of the caeca by *C. hepaticus* may influence gut microbial homeostasis and contribute to disease progression. Our previous study showed that *C. hepaticus* infection alters the caecal microbiota, reducing beneficial short-chain fatty acid–producing bacteria such as *Faecalibacterium* and *Bifidobacterium* [23]. Such dysbiosis may compromise gut health and exacerbate hepatic inflammation during SLD. Limited systemic dissemination was also evident, with detectable titres in spleen from 1 dpi and ovarian follicles from 4 dpi, suggesting potential direct effects on reproductive tissues, like that seen with *Salmonella enterica* invasion [57]. These findings support the acute yet focal nature of SLD and provide a mechanistic explanation for the marked drops in egg production observed during outbreaks [13].

### 4.2. Host Gene Expression Changes and Implications for Egg Production

*C. hepaticus* infection in laying hens shifts hepatic gene expression away from pathways supporting yolk precursor and lipid synthesis and toward stress and immune responses. This pattern suggests a systemic metabolic reallocation that could secondarily impact ovarian function and egg production. *DHCR7*—catalysing the final step in cholesterol biosynthesis for steroid hormone and vitellogenin production—was significantly downregulated [58], and ME1, which supplies NADPH for hepatic fatty-acid and yolk lipid synthesis, was also suppressed, implying reduced lipogenesis and fewer eggs [30,59]. PRLR expression also decreased, likely diminishing prolactin-driven follicle growth and yolk deposition. Together, these changes suggest an infection-induced shutdown of the endocrine and lipid pathways, which normally support egg development, consistent with the marked drops in egg production and egg size reported in *C. hepaticus* infection [60]. Downregulation of PRLR (3 dpi–0.73, 6 dpi–0.97) and DHCR7 (3 dpi–0.73, 6 dpi–0.97) was most pronounced at 6 dpi, coinciding with the highest pathogen load and lesion severity. This pattern suggests a transient metabolic diversion toward immune defence, possibly mediated by cytokine-induced inhibition of steroidogenic and lipogenic pathways [26,61,62]. The time-specific expression changes likely reflect stage-dependent host responses to infection, where dynamic, temporally regulated mechanisms—such as hormone- or cytokine-mediated signalling—modulate hepatic metabolism and egg production during SLD.

Conversely, genes associated with nutrient uptake, calcium regulation, and stress protection were induced. *AVD*—an acute-phase egg-white protein—was strongly upregulated (days 3–7), reflecting inflammatory responses diverting resources from reproduction [31]. VDR increased around day 5, plausibly reflecting a compensatory effort to sustain calcium uptake for eggshell calcification under infection-related stress [32]. VLDLR, the oocyte receptor for VLDL/vitellogenin, rose at days 5–6, suggesting an attempt to preserve yolk-precursor import despite limited supply [35]. VCAN was elevated across all time points, in keeping with extracellular-matrix remodelling during inflammation, and CLU showed late modest induction, consistent with granulosa-cell stress and cytoprotection [39]. These changes indicate a shift from reproductive investment toward stress and immune defence, offering a mechanistic basis for reduced egg production and quality reported during spotty liver disease outbreaks [28,60]. While these results are at the transcript-level, further protein- or metabolite-level analyses (e.g., vitellogenin or yolk lipid quantification) would validate these regulatory trends.

### 4.3. Host Gene Expression Changes Related to Liver Functions

Following *C. hepaticus* infection, the hen liver mounted a pronounced acute-phase inflammatory response. Key positive acute-phase genes, including SAA, ORM1/α1-acid glycoprotein, and HPX, were strongly induced. In chickens, SAA is the dominant acute-phase reactant, rising 100–1000-fold during inflammation [40]. Consistent with this, SAA reached ≈32-fold above control at day 5 (log_2_FC ≈ 5.02), while ORM1 increased about eight-fold, in line with its classification as a moderate acute-phase protein [41]. HPX also increased later in infection (log_2_FC ≈ 1.9 by day 6), reflecting its haem-scavenging and cytoprotective role [42]. CLU, a stress-inducible glycoprotein chaperone, showed modest induction at days 6–7, consistent with its known role in protecting hepatocytes under injury or inflammation [45,46]. Together, these findings demonstrate robust activation of innate immune response genes involved in pathogen recognition and inflammation.

In parallel, genes linked to extracellular matrix remodelling and metabolic homeostasis were altered. VCAN was consistently upregulated across days 3–7, in line with reports of VCAN accumulation during liver fibrosis and stellate cell activation [37,44]. APOA4 rose significantly by day 7, consistent with its hepatoprotective and antioxidant functions [43]. Conversely, two key metabolic genes were suppressed late in infection. PHGDH, the rate-limiting enzyme in serine biosynthesis, was downregulated at 7 dpi; its repression may impair glutathione production and exacerbate oxidative stress [47]. Similarly, CYP1A2, a major detoxification enzyme, was downregulated, consistent with inflammatory suppression of xenobiotic metabolism [48]. These findings suggest that *C. hepaticus* drives hepatic inflammation and metabolic reprogramming at the expense of normal liver function.

We acknowledge that RNA-seq analysis did not include the earliest stages of infection (days 1–2), which limits our understanding of the initial host response to *C. hepaticus* colonisation. Nevertheless, AVD and VCAN emerged as potential biomarkers due to their reproducible transcriptional regulation and known biological roles. Our transcriptomic data showed strong and consistent upregulation of these genes from 3 to 7 dpi, coinciding with the detection of liver lesions, which suggests diagnostic potential. Further investigation of changes in their protein levels in serum could support their use in diagnosing early or asymptomatic infections.

## 5. Conclusions

This study reveals a distinct progression of *C. hepaticus* infection in layer hens, from initial hepatic colonisation to significant biliary amplification, correlating with liver lesion development. For the first time, *C. hepaticus* has been shown to be present in the spleen and ovarian follicles following infection, suggesting that the bacterium may have direct effects on the reproductive tract and immune responses. Changes in gene expression in the liver, including the downregulation of metabolic pathways and the upregulation of acute-phase and tissue remodelling genes like AVD and VCAN, highlight the impact of SLD on liver function and egg production. These insights enhance our understanding of SLD pathogenesis and identify potential biomarkers for monitoring and managing the disease. Targeted interventions focusing on these pathways could improve disease control and reduce economic losses due to SLD.

## Figures and Tables

**Table 1:** Liver lesion development in challenged hens following *C. hepaticus* infection from 1 to 7 days post-infection (dpi).

DPI	Number of Birds with Lesions *	Lesion Score **
1	0	0
2	0	0
3	0	0
4	3	1, 3, 3
5	4	4, 4, 1, 3
6	6	3, 4, 1, 3, 4, 4
7	6	3, 3, 3, 4, 3, 3

* Six infected birds were necropsied at each time point. ** Lesions were scored according to the number of visible miliary foci on the liver surface: 0 = no lesions; 1 = 1–5 spots; 2 = 6–20 spots; 3 = 21–100 spots; 4 = 101–1000 spots; 5 ≥1000 spots [12].

**Table 2:** Differentially expressed genes associated with egg production in *C. hepaticus*—infected hens (FDR < 0.05). Each gene’s primary function in laying hens is noted, along with days post-infection (dpi) at which significant differential expression was detected, the direction of regulation, and the magnitude of change (log_2_ fold change).

Gene	Function	Days (Dpi)/Log_2_FC	Regulation
**PRLR**	Mediates prolactin’s effects on follicle development and yolk precursor synthesis [26,27,28].	D3/−0.73; D6/−0.97	Down
**DHCR7**	Final enzyme in cholesterol synthesis (needed for steroid hormone production for yolk development) [29].	D3/−0.73; D6/−0.97	Down
**ME1**	Generates NADPH for fatty-acid synthesis in liver (critical for yolk lipid/vitellogenin production) [30].	D4/−1.18; D6/−1.09	Down
**AVD**	Oviduct egg-white protein; acute-phase reactant diverting resources from egg formation [31].	D3/3.87; D4/5.32; D5/7.23; D6/5.49; D7/6.16	Up
**VDR**	Regulates calcium absorption and eggshell calcification (calcium homeostasis in intestine, shell gland, etc.) [32,33,34].	D5/7.19; D7/3.45	Up
**VLDLR**	Oocyte receptor for yolk precursors (VLDL/vitellogenin) required for yolk uptake into developing eggs [35,36].	D5/0.91; D6/0.87	Up
**VCAN**	Extracellular matrix proteoglycan involved in follicular tissue remodelling [37,38].	D3/1.27; D4/1.19; D5/1.69; D6/1.87; D7/1.41	Up
**CLU**	Ovarian glycoprotein (in granulosa cells) aids yolk protein transport and protects cells from stress [39].	D6/0.84; D7/0.09	Up

**Table 3:** Liver function-related genes differentially expressed in *C. hepaticus*–infected hen livers. Listed are major hepatic functions associated with each gene, days post-infection with significant expression changes, direction of regulation, and observed log_2_fold changes (FC).

Gene	Key Function in Liver	Days (Dpi)/Log_2_FC	Regulation
**SAA**	Major acute-phase protein produced by liver; marker of systemic inflammation [40].	D3/1.43; D4/3.99; D5/5.02; D6/4.93; D7/3.75	Up
**ORM1**	Acute-phase glycoprotein; immunomodulator that increases during inflammation [41].	D3/1.35; D4/3.02; D5/2.58; D6/2.09; D7/1.99	Up
**HPX**	Haem-binding protein that protects against haem-induced oxidative damage [42].	D4/1.56; D5/1.60; D6/1.93; D7/1.24	Up
**APOA4**	Lipoprotein involved in lipid transport and metabolism; has roles in antioxidant response [43].	D7/1.50	Up
**VCAN**	Extracellular matrix proteoglycan; rises during liver inflammation and fibrosis [37,44].	D3/1.27; D4/1.19; D5/1.69; D6/1.87; D7/1.41	Up
**CLU**	Stress-induced chaperone; protects liver cells from oxidative stress/apoptosis [45,46].	D6/0.84; D7/0.09	Up
**PHGDH**	Enzyme for de novo serine synthesis (supports glutathione and phospholipid production) [47].	D7/−0.90	Down
**CYP1A2**	Key detoxification enzyme in liver (metabolises drugs and toxins) [48].	D7/−1.16	Down

## Data Availability

The raw sequence data generated in this study are available from the NCBI SRA database under accession number PRJNA1338597.

## Abbreviations

The following abbreviations are used in this manuscript:

DHCR7	7-dehydrocholesterol reductase
APOA4	Apolipoprotein A-IV
AVD	Avidin
CLU	Clusterin
CYP1A2	Cytochrome P450 1A2
GRCg7b	Gallus gallus reference genome assembly (Ensembl)
GTF	Gene Transfer Format (annotation)
HPX	Haemopexin
ME1	Malic enzyme 1
ND	Not detected
ORM1	Orosomucoid-1
PHGDH	Phosphoglycerate dehydrogenase
PRLR	Prolactin receptor
qPCR	Quantitative polymerase chain reaction
RIN	RNA Integrity Number
RNA-seq	RNA sequencing
SAA	Serum amyloid A
SEM	Standard error of the mean
SLD	Spotty liver disease
VCAN	Versican
VLDL	Very low-density lipoprotein
VLDLR	Very low-density lipoprotein receptor
VDR	Vitamin D receptor
